# A prognostic model of non small cell lung cancer based on TCGA and ImmPort databases

**DOI:** 10.1038/s41598-021-04268-7

**Published:** 2022-01-10

**Authors:** Dongliang Yang, Xiaobin Ma, Peng Song

**Affiliations:** 1 Department of General Education, Cangzhou Medical College, Cangzhou, 061001 China; 2 Department of Respiratory Medicine, Shandong Provincial Hospital Affiliated to Shandong First Medical University, Jinan, 252200 China

**Keywords:** Cancer, Lung cancer, Non-small-cell lung cancer

## Abstract

Bioinformatics methods are used to construct an immune gene prognosis assessment model for patients with non-small cell lung cancer (NSCLC), and to screen biomarkers that affect the occurrence and prognosis of NSCLC. The transcriptomic data and clinicopathological data of NSCLC and cancer-adjacent normal tissues were downloaded from the Cancer Genome Atlas (TCGA) database and the immune-related genes were obtained from the IMMPORT database (http://www.immport.org/); then, the differentially expressed immune genes were screened out. Based on these genes, an immune gene prognosis model was constructed. The Cox proportional hazards regression model was used for univariate and multivariate analyses. Further, the correlations among the risk score, clinicopathological characteristics, tumor microenvironment, and the prognosis of NSCLC were analyzed. A total of 193 differentially expressed immune genes related to NSCLC were screened based on the "wilcox.test" in R language, and Cox single factor analysis showed that 19 differentially expressed immune genes were associated with the prognosis of NSCLC (*P* < 0.05). After including 19 differentially expressed immune genes with *P* < 0.05 into the Cox multivariate analysis, an immune gene prognosis model of NSCLC was constructed (it included 13 differentially expressed immune genes). Based on the risk score, the samples were divided into the high-risk and low-risk groups. The Kaplan–Meier survival curve results showed that the 5-year overall survival rate in the high-risk group was 32.4%, and the 5-year overall survival rate in the low-risk group was 53.7%. The receiver operating characteristic model curve confirmed that the prediction model had a certain accuracy (AUC = 0.673). After incorporating multiple variables into the Cox regression analysis, the results showed that the immune gene prognostic risk score was an independent predictor of the prognosis of NSCLC patients. There was a certain correlation between the risk score and degree of neutrophil infiltration in the tumor microenvironment. The NSCLC immune gene prognosis assessment model was constructed based on bioinformatics methods, and it can be used to calculate the prognostic risk score of NSCLC patients. Further, this model is expected to provide help for clinical judgment of the prognosis of NSCLC patients.

## Introduction

Globally, due to high-risk factors, such as smoking, radon, occupational exposure, traffic exhaust, and air pollution, lung cancer has become the leading cause of cancer-related deaths, and it is also a major global health problem that is currently attracting widespread attention^1^. Non-small cell lung cancer (NSCLC) accounts for 85% of lung cancer diagnoses. Approximately 50% of NSCLC patients are in stage IV when they are detected, and their 5-year survival rate is less than 10%^2^. In recent years, immune checkpoint inhibitors (ICIs) targeting programmed cell death 1 (PD-1) or its ligands (PD-L1) have been developed, which has caused significant progress in the treatment and overall management of locally advanced and advanced NSCLC^3^. The role of abnormal expression of tumor immune-related genomes in tumor immune evasion has become a new direction in tumor research. Abnormal immune genomes have an important impact on patients with ovarian cancer, gastric cancer, liver cancer, and kidney cancer^4^. However, there is no relevant report on how an abnormal genome affects NSCLC. In addition, a variety of molecular markers are used to predict the prognosis of NSCLC, but they have not yet been widely recognized. Therefore, it is necessary to explore the genes related to the prognosis of NSCLC at the molecular level, and the construction of genetic models related to the prognosis of NSCLC has a strong clinical significance. Therefore, this study is based on the TCGA and ImmPort data sets to explore immune gene expression and immune cell differential analysis, and combine its clinicopathological characteristics and immune gene characteristics to construct a prognostic model of NSCLC, it is expected to have certain guiding significance for the treatment of NSCLC.

## Materials and methods

### Data download

The transcriptomic data and clinical data of NSCLC patients in TCGA-LUAD and TCGA-LUSC were downloaded through the Genome Data General Database (GDC) data portal, and 929 clinical data were obtained. Immune gene data were downloaded through the ImmPort data portal, and 2498 immune-related genes were obtained. The transcription factor data were downloaded from the Cistorm website.

### Differential expression analysis

#### Differential gene expression analysis

The Wilcoxon test in R software was used to analyze the differences in all transcriptomic data, and to screen out genes with significant differences in expression between normal tissues and tumor tissues. The screening criteria were |logFC|> 1, FDR < 0.05.

#### Analysis of immune gene differences

Differential genes were combined with the acquired immune gene data and analyzed in R software to screen out differential immune genes from all differential genes.

### Establishment and evaluation of the immune gene prognosis model

The expression of immune genes was combined with survival time and survival status, survival analysis of differential immune genes and clinical survival time was conducted, and immune genes that could affect the prognosis of NSCLC were determined. Based on these genes, an immune gene prognostic model was constructed. Receiver operating characteristic (ROC) and risk scoring curves were drawn in R software to evaluate the effectiveness of this model.

### Correlation analysis between risk score and immune cells

Immune genes related to the prognosis were combined with clinical data, and the patient's risk score was calculated based on the immune gene prognosis model. Correlation analysis was performed between risk score and immune cells infiltrated by the tumor microenvironment (immune cell data were downloaded from the TIMER immune cell infiltration database).

### Statistical methods

R 3.6.0 software (https://mirrors.tuna.tsinghua.edu.cn/CRAN/) was used for statistical analysis and graph drawing. The wilcox.test was used to screen differential genes. The "ggplot" package was used for graph drawing, and the "survival" package was used for single-factor and multi-factor Cox proportional regression model screening and to establish the multiple gene prognosis model. The "survival ROC" package was used to calculate the ROC curve to evaluate the effectiveness of the model and the area under the curve (AUC). The statistical inference level was set at two-sided α = 0.05.

## Results

### Screening of differentially expressed immune genes

**Figure 1:**
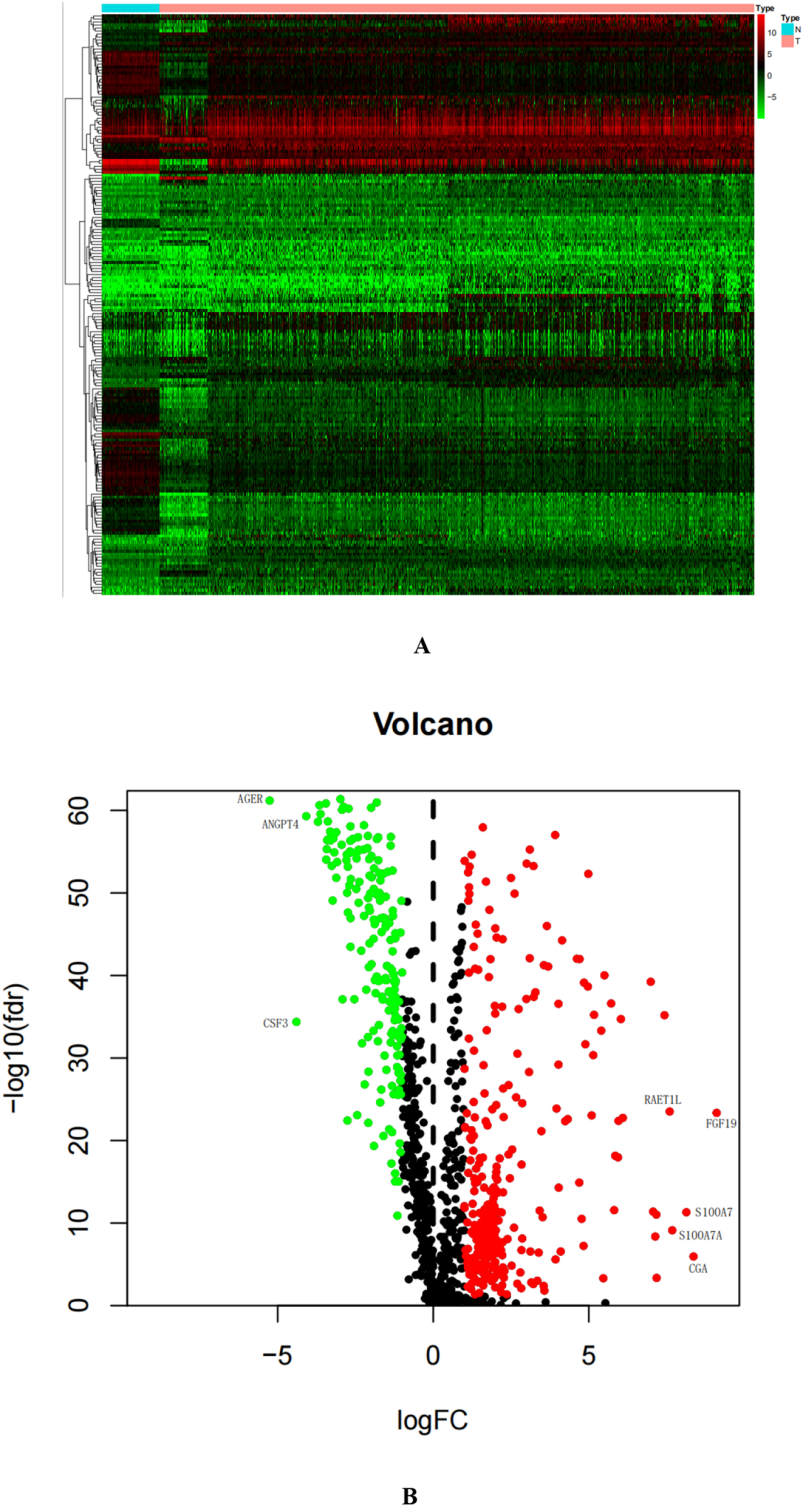
A heat map (**A**) and a volcano map (**B**) of differential immune genes. The heat map abscissa represents the sample: the blue area represents normal tissue and the red area represents tumor tissue; the ordinate represents the gene. On the volcano map, the green area represents the downregulated differential genes and the red area represents the upregulated differential genes.

Data of the TCGA database containing 1128 non-small cell lung cancer samples and 110 normal tissues were downloaded. Differential expression analysis screened a total of 2875 differential genes (FDR < 0.01, |log2FC|> 1), of which 2317 differential genes were highly expressed and expression of 557 differential genes was low. A total of 2498 tumor-related immune genes were downloaded from the ImmPort database. In the R language, immune genes and all differentially expressed genes were intersected and a total of 193 differential immune genes related to NSCLC were obtained, of which 121 differential immune genes were highly expressed and 72 differentially expressed genes were lowly expressed. The R-ggplot package (version: 3.3.5) was used to draw a heat map (Fig. 1A), and the R-pheatmap package (version: 1.0.12) was used to draw a volcano map (Fig. 1B).

### Transcriptional regulatory network mapping

**Figure 2:**
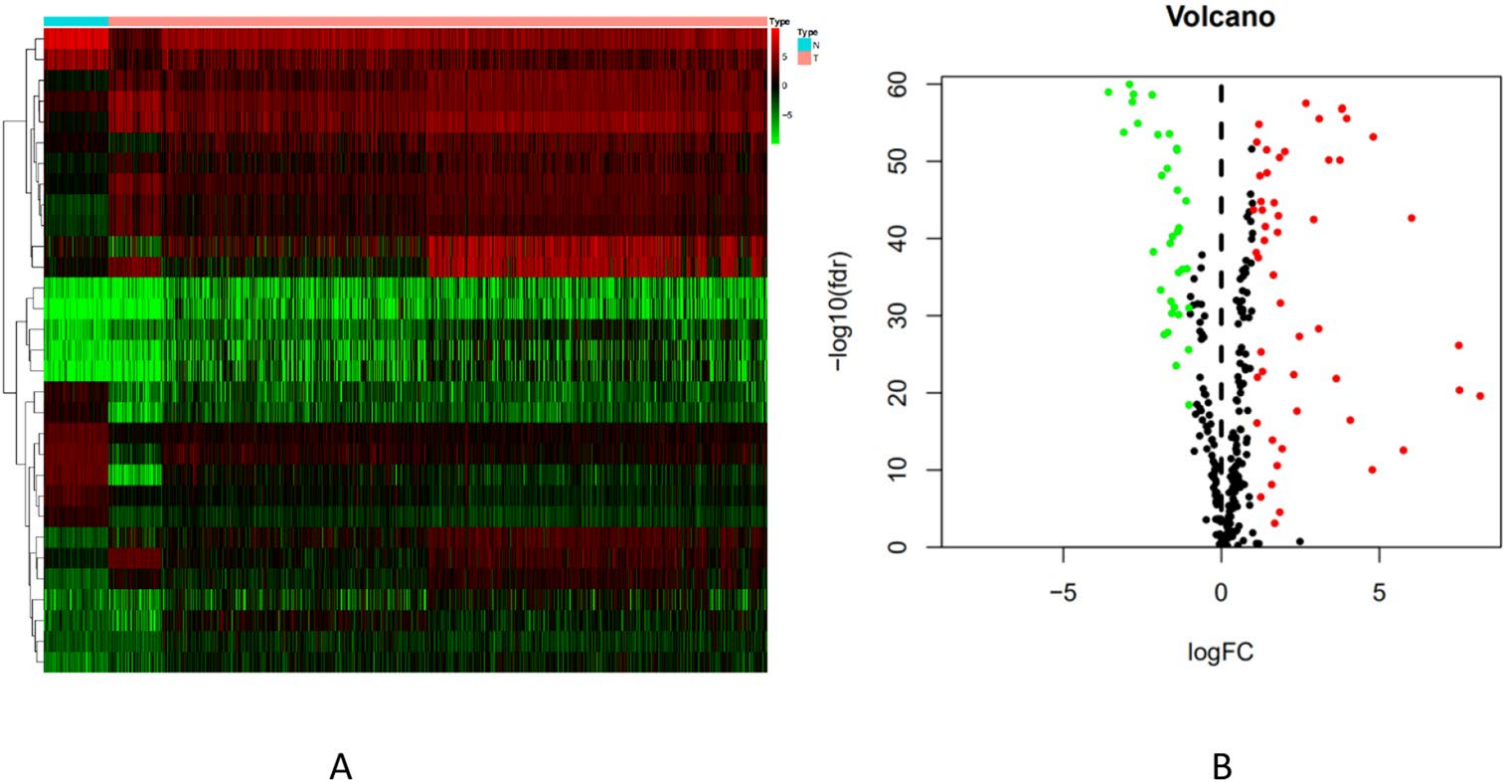
Heat map and volcano map of differentially expressed TFs of non-small cell lung cancer. **A** heat map of differentially expressed TFs of non-small cell lung cancer, red represents high expression, blue represents low expression; **B** volcano map of TFs of non-small cell lung cancer. The X-axis is log FC, and the larger the absolute value is, the larger the corrected *P* value is, indicating the larger the multiple of the difference is. The Y-axis is the corrected *P* value, and the larger the logarithm of log10 is, indicating the more significant the difference is.

**Figure 3:**
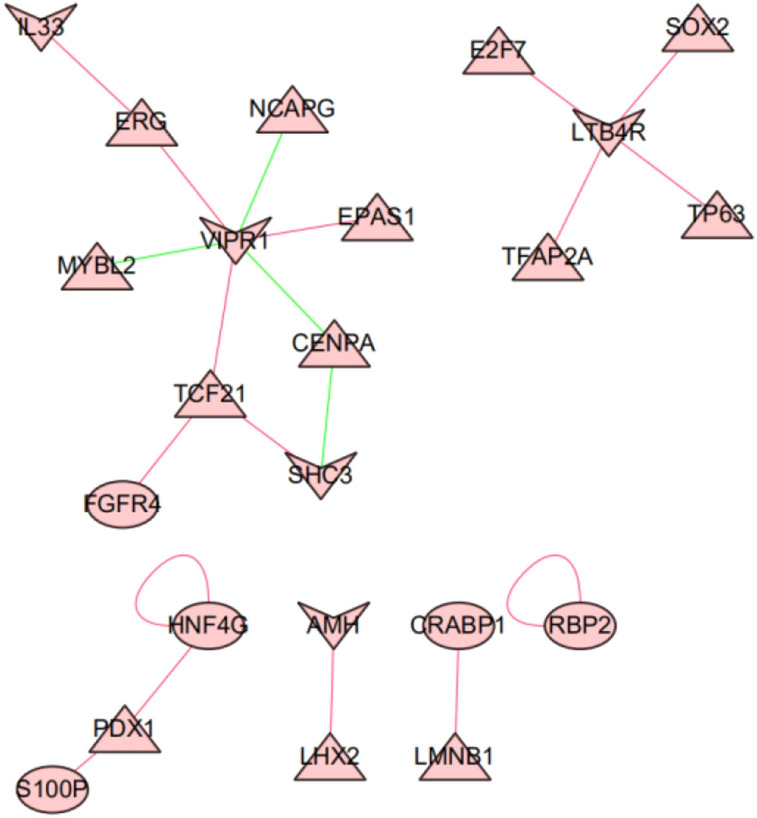
Transcriptional factors and immune gene regulatory network (Triangles represent transcription factors, circles represent high-risk immune genes, and cones represent low-risk immune genes; The red line represents positive regulation, and the blue line represents negative regulation).

A total of 318 transcription factors (TFs) downloaded from the Cistrome Cancer database and 2875 differentially expressed genes were crossed to obtain 83 differentially expressed TFs, of which 50 TFs were up-regulated and 33 TFs were down-regulated (Fig. 2A, B). The correlation between immune-related genes and differentially expressed TFs was further analyzed by Pearson correlation test, and the intersection group with correlation coefficient > 0.4 and *P* < 0.01 was screened out. Cytoscape was used to draw the immune factors and transcriptional gene regulatory network (Fig. 3). VIPR1 (low-risk immune genes) is negatively regulated by NCAPG, MYBL2, CENPA and positively regulated by ERG, EPAS1, TCF21. SHC3 is negatively regulated by CENPA and positively regulated by TCF21. All the other genes were positively regulated.

### Establishment of an immune gene prognostic model for NSCLC

**Figure 4:**
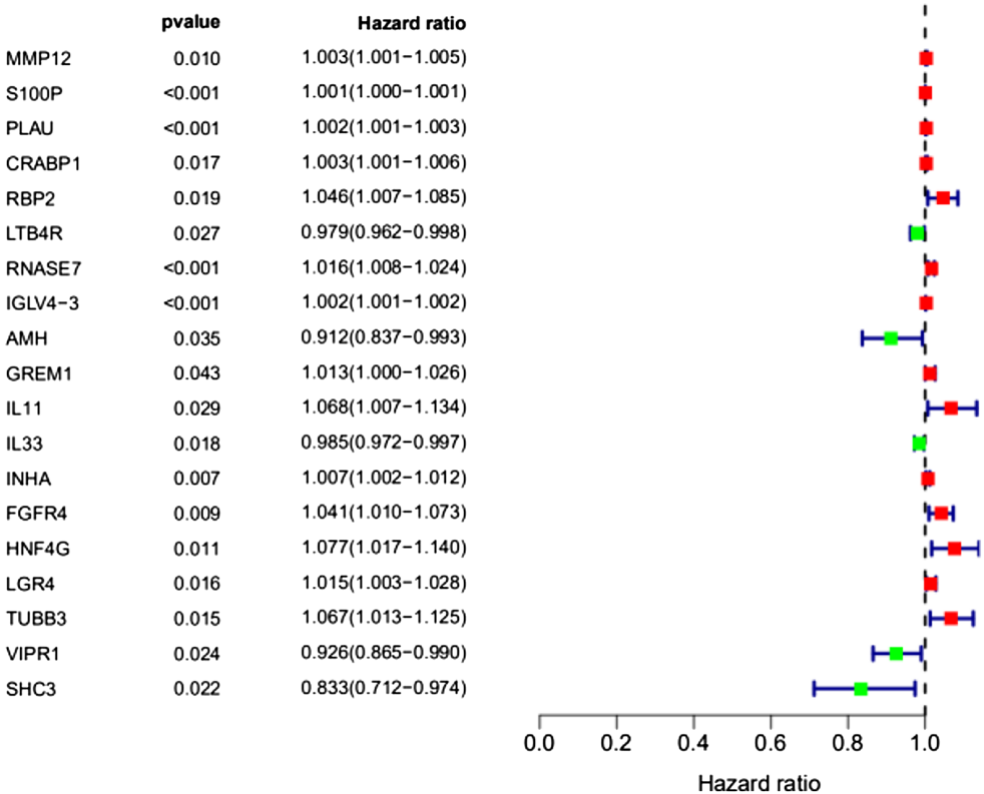
Forest map of 19 differentially expressed immune genes in the univariate Cox regression model.

Univariate regression analysis was performed on 193 differential immune genes related to NSCLC. The results showed that 19 differential immune genes were significantly related to the overall survival rate of NSCLC (*P* < 0.05) (Fig. 4).

**Figure 5:**
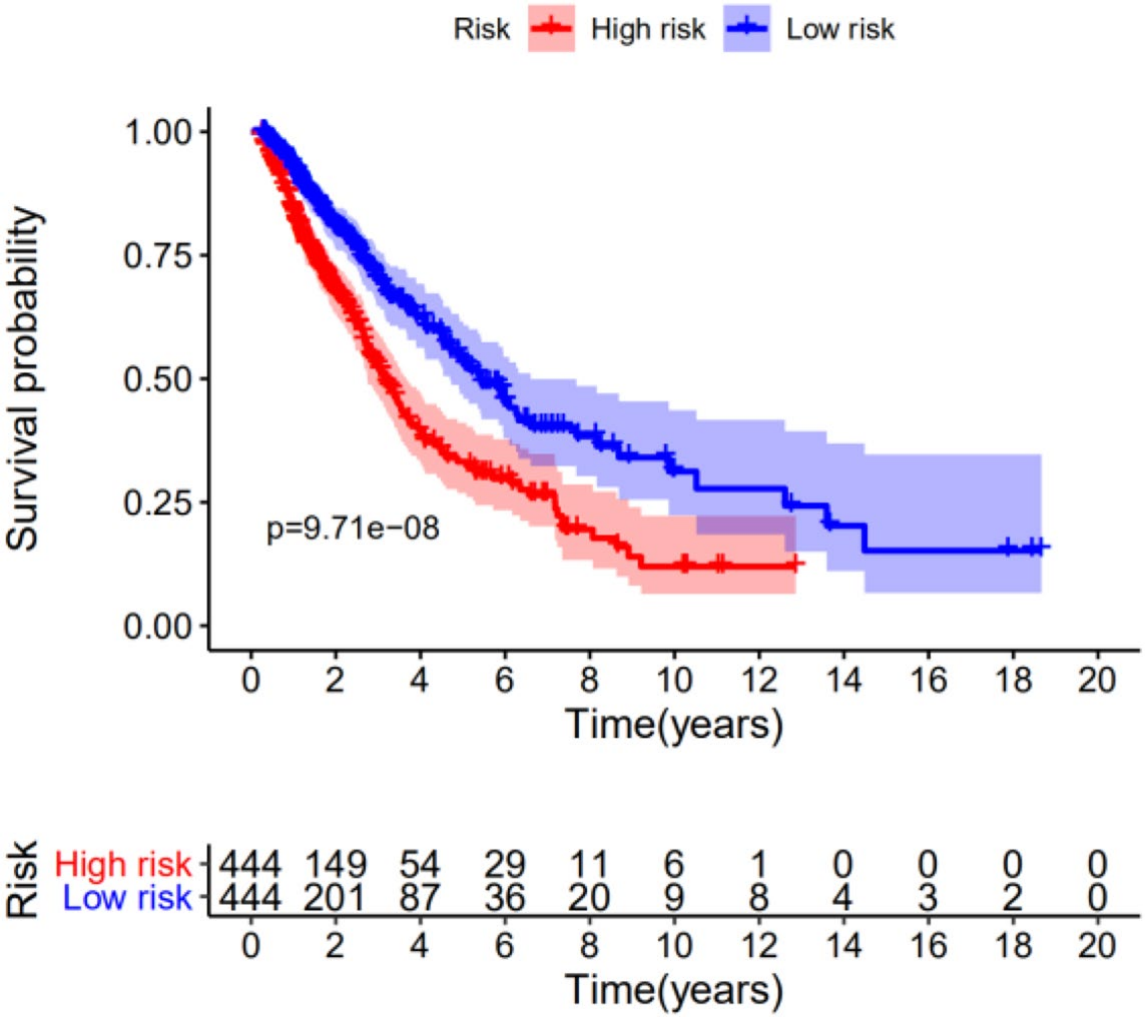
Kaplan–Meier survival analysis of non-small-cell lung cancer patients by risk stratification.

**Figure 6:**
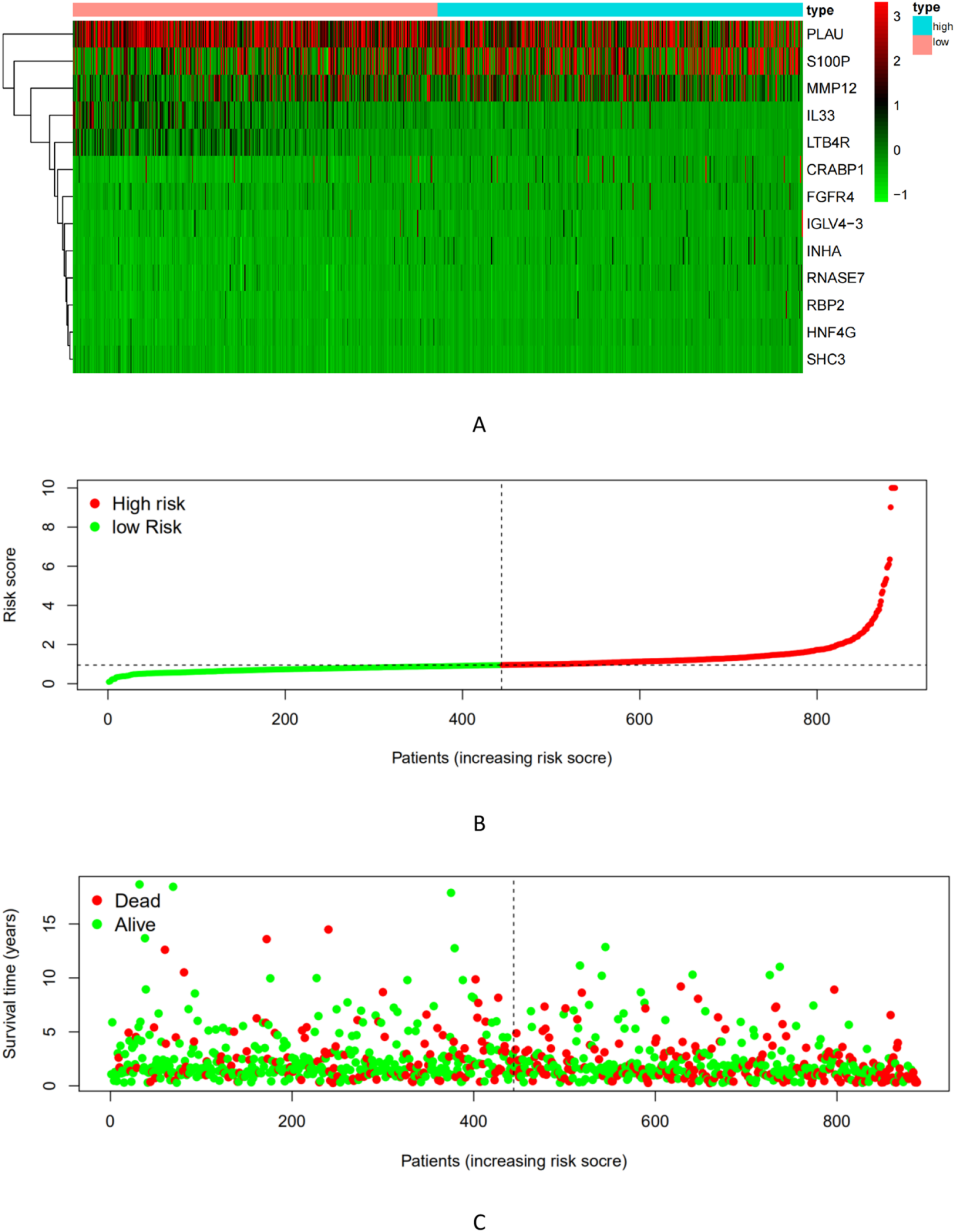
Risk score curve and survival heat map. (**A**) survival heat map, with the increase of risk score, the expression of immune genes increased; (**B**) risk score curve, from left to right, the patient's risk score increased gradually; (**C**) point of survival chart (With the increase of patients'risk value, more patients died).

**Figure 7:**
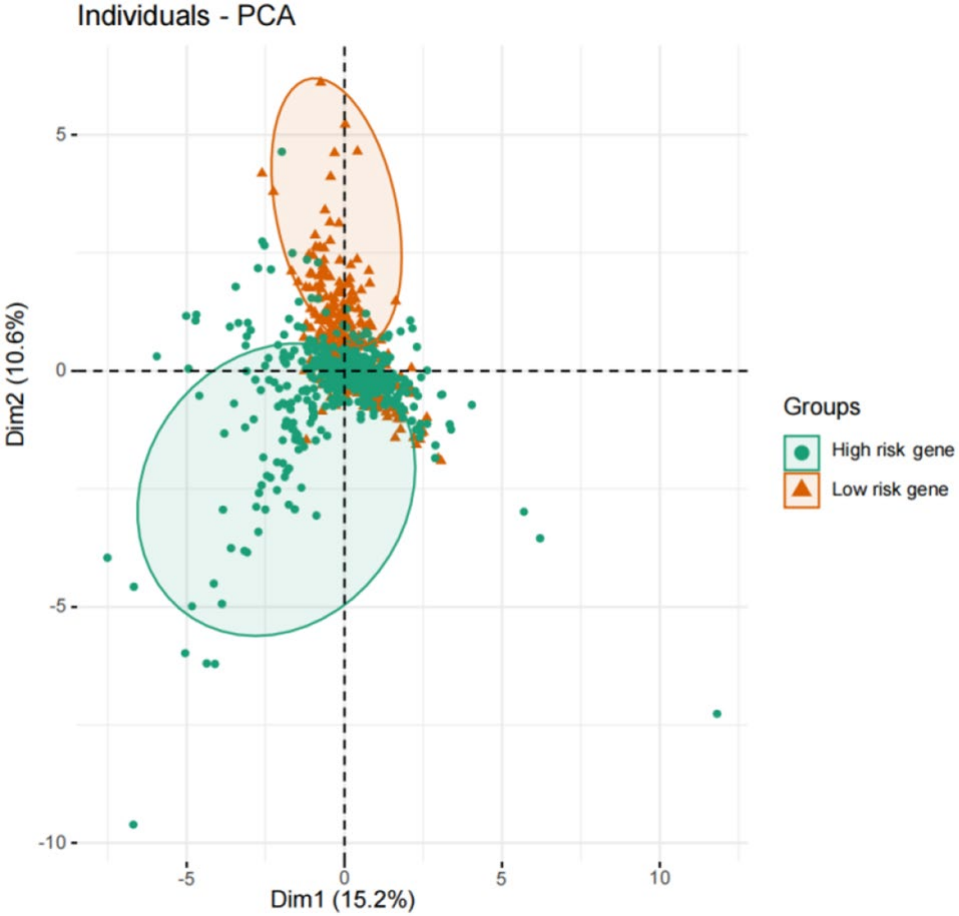
Principal component analysis plot using expression values at 13 selected immune genes.

These 19 differential immune genes with *P* < 0.05 were selected for the Cox multivariate analysis, and the NSCLC prognosis model containing 13 differential immune genes was obtained: Risk Score = MMP12 × 0.0022 + PLAU × 0.0023 + S100P × 0.003 + CRABP1 × 0.0036 + RBP2 × 0.0531 + LTB4R × (-0.0255) + RNASE7 × 0.0174 + IGLV4-3 × 0.0017 + IL33 × (-0.014) + INHA × 0.0053 + FGFR4 × 0.0443 + SHC3 × (-0.1775) + HNF4G × 0.0516. From the risk score, LTB4R, IL33 and SHC3 are the immune genes that are beneficial for the prognosis of NSCLC. Using the median of Risk Score (RS) (0.9506237) as the boundary value, the RS was divided into high risk and low risk groups, and patients were sorted according to risk scores from low to high. The risk score curves (Fig. 5) and survival heat maps (Fig. 6A) were drawn. The patient was used as the abscissa to plot the RS and survival time. It was noted that as the RS score increased, the immune gene expression content increased (Fig. 6B); and as the RS increased, the patient's survival time shortened and the number of deaths increased significantly (Fig. 6C). Use the R package "princomp" to perform principal component analysis (PCA) on 13 immune genes, it can be seen that the high-risk genome and the low-risk genome are clearly divided into two discontinuous groups (Fig. 7).

### COX survival analysis and prognostic model evaluation

**Figure 8:**
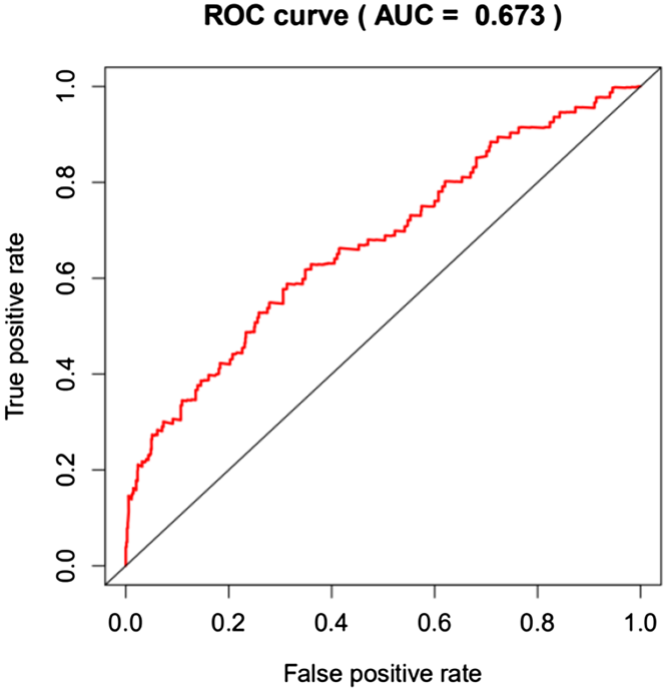
ROC curve of multivariate Cox analysis model.

After using the R-survival package to perform COX survival analysis in the high-risk and low-risk groups, the results showed that the 5-year survival rate in the high-risk group was 32.4%, and the 5-year survival rate in the low-risk group was 53.7%. The difference was statistically significant (*P* < 0.01). In order to further verify the accuracy of the prognostic evaluation model, we used the R-survival ROC package to draw the model ROC curve (Fig. 8), and the results showed an AUC = 67.3%. This finding suggested that the risk assessment model had better sensitivity and specificity in predicting the prognosis of NSCLC.

**Figure 9:**
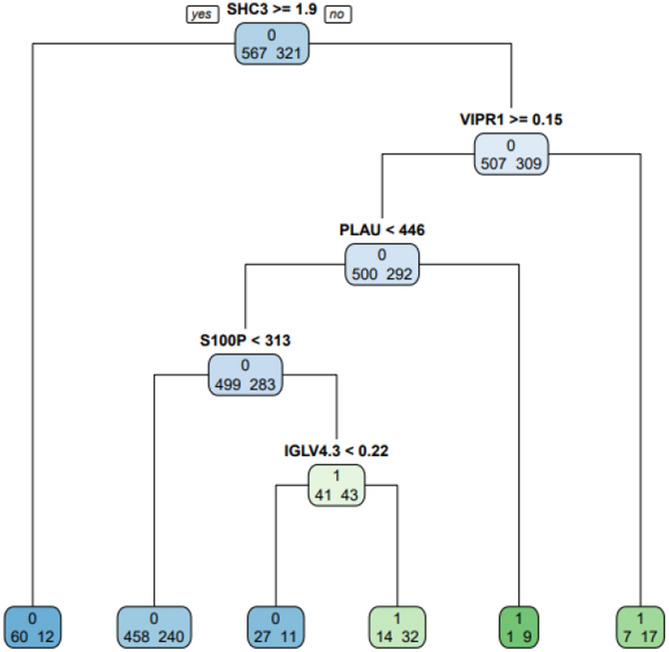
Immune gene prediction model (decision tree algorithm).

**Figure 10:**
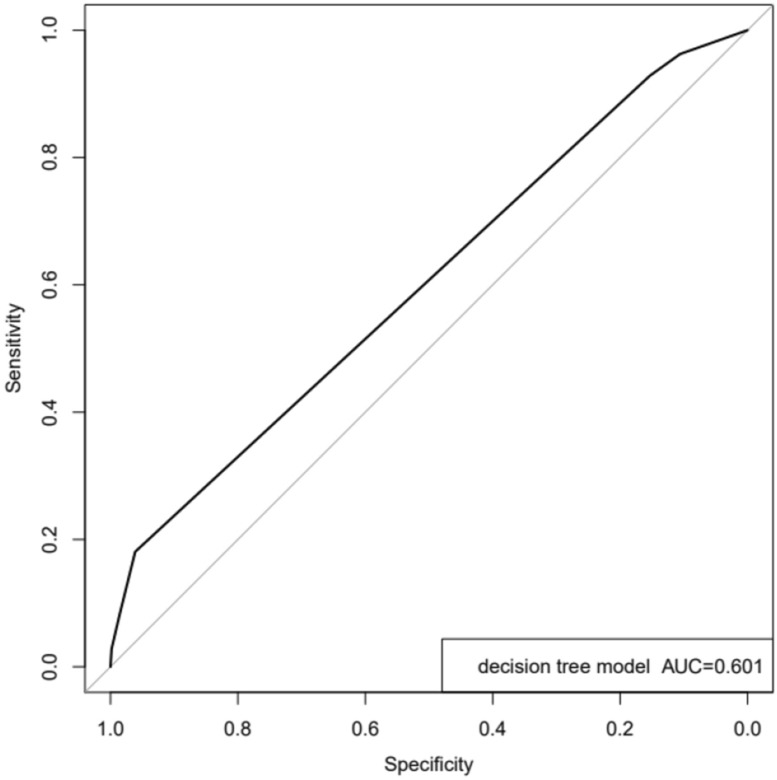
ROC curve of decision tree algorithm model.

In order to verify whether the machine learning modeling algorithm is better than the traditional COX regression analysis, We use the decision tree algorithm in machine learing to build a new model. Using the rpart package of R version 4.0.2 to model the original data decision tree (Fig. 9), it was found that the area under the ROC curve of the decision tree model was 0.601 (Fig. 10), which was lower than the model established by the original cox regression area under the curve. Therefore, the model established by cox proportional hazard regression is the final predictive model.

### An independent prognostic factor in NSCLC

**Figure 11:**
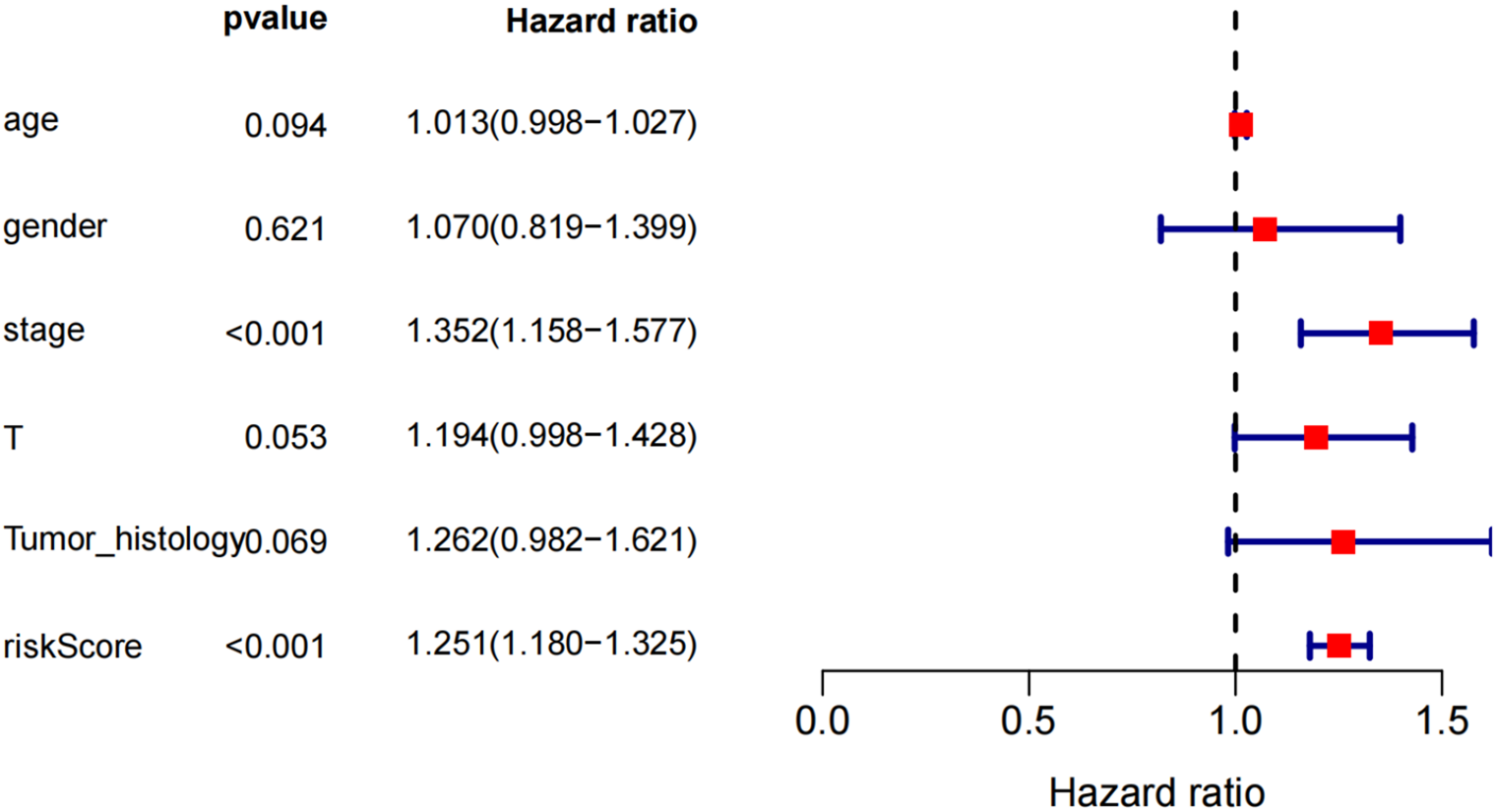
Cox multivariate regression analysis.

In order to further verify whether the prediction model can independently predict the prognosis of NSCLC, various parameters in the clinical data downloaded from the TCGA database were used as independent variables, and the patient's survival time was used as the dependent variable to perform Cox factor regression analysis. The results suggested that the risk score was an independent risk factor affecting the prognosis of NSCLC (*P* < 0.05) (Fig. 11).

### Validation of the prognostic value of risk score using the GEO datasets

**Figure 12:**
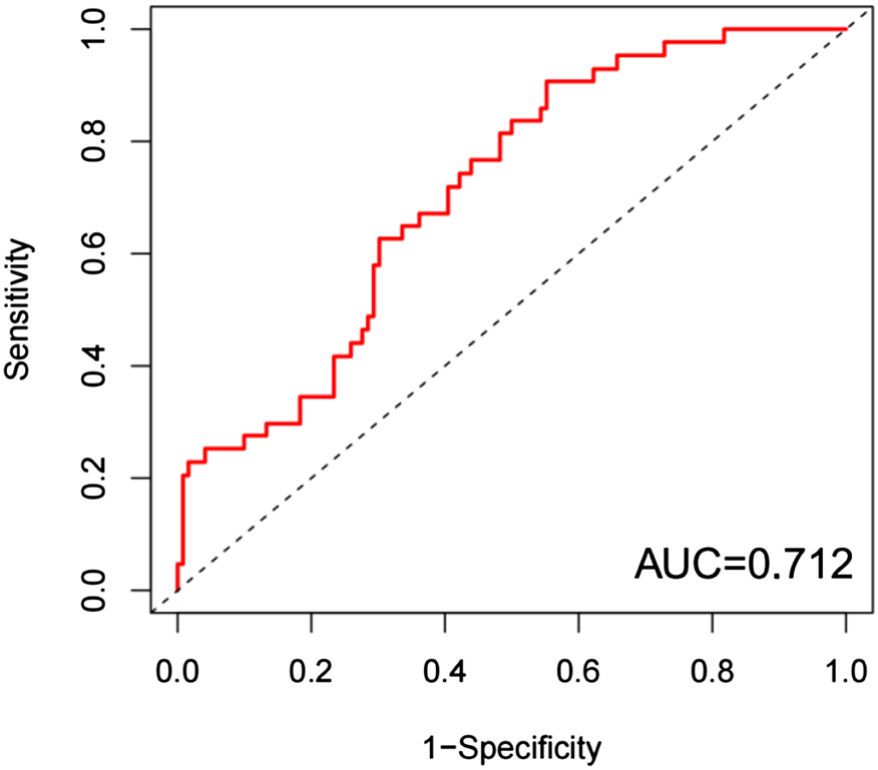
ROC curve of multivariate Cox analysis model in GSE68465 database.

**Figure 13:**
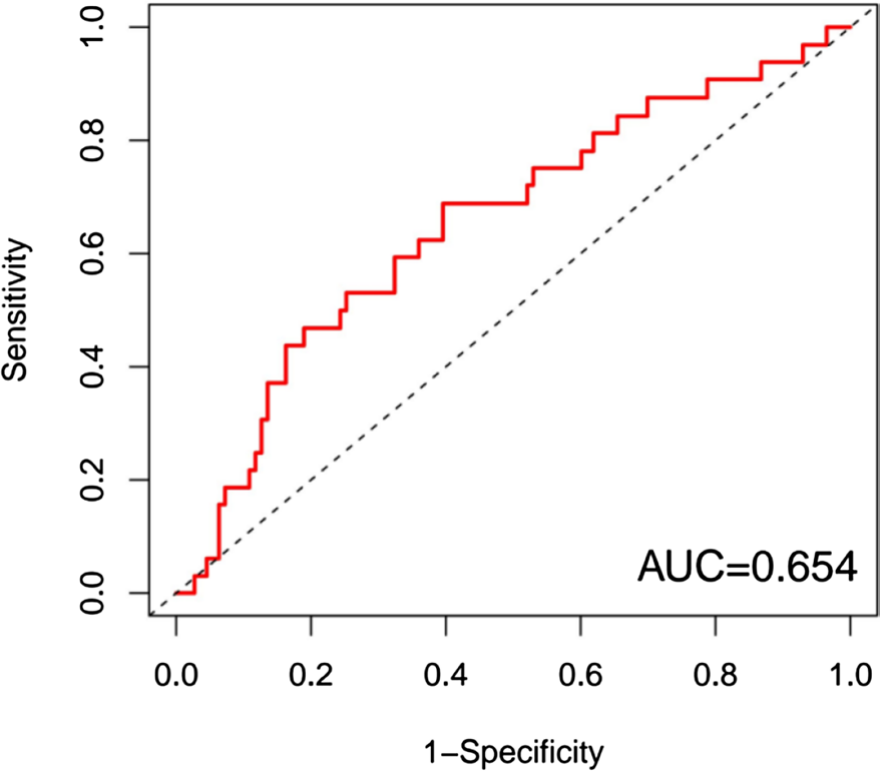
ROC curve of multivariate Cox analysis model in GSE101929 database.

Independent validations were conducted using the GEO datasets to further test the prognostic value of risk score. The same method was used to generate a risk score for risk stratification of NSCLC patients in GSE68465 and GSE101929. In order to further verify the accuracy of the prognostic evaluation model in GEO database, we used the R-survival ROC package to draw the model ROC curve (Figs. 12 and 13), and the results showed an AUC = 71.2% (GSE68465) / 65.4% (GSE101929). This result can confirm that the 13-gene NSCLC prediction model still performs well in the GEO database.

### The relationship between risk score and immune cell infiltration

**Figure 14:**
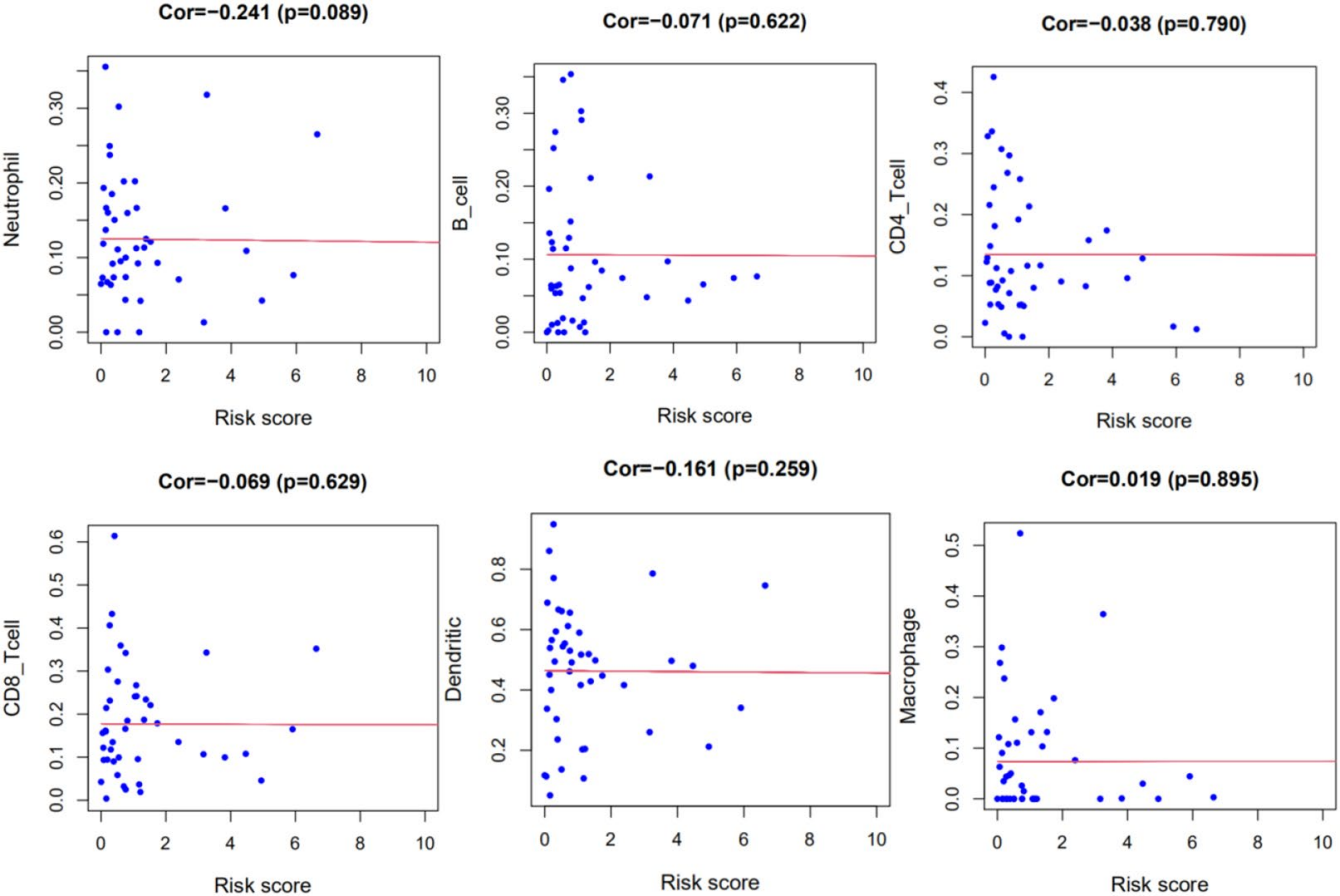
Correlation analysis between risk score and immune cell infiltration in tumor microenvironment.

We also analyzed the relationship between risk score and immune cell infiltration in the tumor microenvironment. The results showed that the degree of neutrophil infiltration had a certain correlation with the risk score (*P* = 0.089), but there was no statistically significant difference. There was no correlation with B cells, CD4 + T cells, CD8 + T cells, dendritic cells, and macrophages (Fig. 14).

## Discussion

In the past decade, it has been recognized that the occurrence and development of tumors should not only be attributed to the internal genetic background of cancer cells, but it is also related to the interaction of various systems in the body^5^, especially the immune system^6^. Immune-related cells and factors are involved in the entire process of tumorigenesis, proliferation, and development^7,8^. Therefore, it is necessary to explore the characteristics of immune-related molecules and evaluate the function of immune genes in lung cancer^9^. In this study, we sorted out 2498 immune-related genes in TCGA mRNA in 929 NSCLC patients and further performed COX univariate analysis of 193 immune-related genes, and we found that 19 differential genes were significantly related to the prognosis of NSCLC patients. COX multivariate analysis yielded a NSCLC multivariate prognostic risk model containing 13 differential genes, and the effectiveness of the model was verified by Kaplan–Meier and ROC curves. Among these genes, MMP12, PLAU, S100P, CRABP1, RBP2, RNASE7, IGLV4-3, INHA, FGFR4, and HNF4G may be immune-related genes that promote tumorigenesis; and LTB4R, IL33, and SHC3 may be immune-related genes that inhibit tumorigenesis. The immune gene risk score and clinicopathological characteristics were included in the univariate and multivariate Cox regression analyses of the prognosis of NSCLC. The results suggest that the immune gene risk score is an independent predictor of the prognosis of NSCLC. In view of their important role in the prognostic evaluation of NSCLC, these genes play an important role in the occurrence and development of NSCLC, and they may become new targets for precision treatment of NSCLC, which are worthy of an in-depth study^10^.

More and more evidence shows that abnormally expressed genes can be used as prognostic markers for NSCLC^11^. SD DEr have identified and verified 15 gene characteristics (ATP1B1, TRIM14, FAM64A, FOSL2, HEXIM1, MB, L1CAM, UMPS, EDN3, STMN2, MYT1L, IKBKAP, MLANA, MDM2, ZNF236) that affect the prognosis of NSCLC^12^. Shukla et al. divided the TCGA RNA sequencing data into training and validation cohorts, based on 4 gene characteristics (RHOV, CD109, FRRS1 and long non-coding RNA (lncRNA) genes LINC00941) divide LUAD patients into high-risk and low-risk survival groups^13^. In another study, 20 gene characteristics based on TCGA data can predict the OS of NSCLC, combined with a comprehensive analysis of differentially expressed genes in the GEO data set (GSE85841), including four of FUT4, SLC25A42, IGFBP1 and KLHDC8B Genes can predict OS (AUC of prognostic score 20 genes = 0.615, AUC of prognostic score 4 genes = 0.5731)^14^. Recently, Xie et al. used DE genes in the TCGA and GEO datasets to construct a weighted gene co-expression network, and found that 6 gene features (RRAGB, RSPH9, RPS6KL1, RXFP1, RRM2, and RTL1) can be used for prognostic stratification of lung adenocarcinoma (the area under ROC curve (AUC) was 0.776 in predicting the 10-year survival of NSCLC patients)^15^. Our data shows that the sensitivity of the prediction model is 0.710, the specificity is 0.687, the AUC of prognostic score = 0.673. The prediction accuracy is higher than the data of Zhao K et al., and is similar to the prediction effectiveness of the model of Xie et al.

LTB4R is the first discovered protective gene for NSCLC, and IGLV4-3 is the first discovered harmful gene for NSCLC. The other 11 differential immune-related genes in this prognostic assessment model have been rarely reported in NSCLC. MMP12 is one of the zinc-dependent proteolytic enzymes, which plays a vital role in all aspects of tumor progression (such as tumor angiogenesis and metastasis)^16,17^. Klupp et al. have reported that serum MMP12 levels are a negative prognostic marker in colon cancer patients^18^. In addition, MMP12 polymorphisms are associated with a higher risk of lung cancer^19–21^. Lv FZ et al. found that high expression of MMP12 is related to pathological staging and tumor metastasis of lung adenocarcinoma, indicating that MMP12 may be a promising target for the treatment of lung adenocarcinoma^22^. According to the reports, RNASE7 and PLAU are unfavorable prognostic factors for NSCLC^23,24^. S100P is a pleiotropic tumor-promoting factor. According to the reports, in addition to promoting tumor migration, invasion, and metastasis, S100P also enhances cell proliferation by up-regulating cyclin D1 and CDK2^25^ and confers chemoresistance by binding and inactivating p53^26,27^. Some studies have shown that CRABP1 expression is abnormal in NSCLC and is significantly associated with distant lymph node metastasis. A total of 42% of NSCLC samples have shown elevated CRABP1mRNA levels, which may be related to the transfer of NSCLC^28^. RBP2 is overexpressed in human lung cancer tissues and is necessary for lung cancer cell proliferation, movement, migration, invasion, and metastasis. These capabilities have been further proved to be regulated by the deethylase and DNA binding activity of RBP2. RBP2 directly binds to the integrin b1 (ITGB1) promoter and is involved in tumor migration and invasion^29^. Another study showed that RBP2 regulates the expression of n-cadherin and snails by activating Akt signaling^30^. In addition, ITGB1 and Akt signaling are significantly related to tumor angiogenesis^31,32^. These results also indicate that RBP2 promotes tumor angiogenesis^33,34^. Genetic polymorphisms and abnormal levels of IL33 are closely related to lung cancer^35,36^. Mei LJ also demonstrated the protective effect of IL-33 alleles on lung cancer^37^. Wang JJ conducted a comprehensive review, meta-analysis, and evaluation of the strength of evidence on published studies on lung cancer candidate genes. Among these studies, 2910 gene variants in 754 different genes or chromosomal loci were eligible for inclusion. A major meta-analysis of 246 variants of 138 different genes found that FGFR4rs351855 is significantly associated with the cumulative epidemiological sensitivity of lung cancer^38^. Li R et al. identified SHC3 and IL33 immune genes as independent prognostic factors for predicting the survival of NSCLC patients^39^. Hepatocyte nuclear factor 4 (HNF4) belongs to the orphan nuclear receptor superfamily^40^. Compared with the adjacent normal lung tissue, the expression of HNF4G is significantly up-regulated in lung cancer tissues. The expression level of HNF4G is related to the tumor size and overall survival rate. Genome set enrichment analysis and biological function determination have proved that HNF4G can exert a carcinogenic effect by promoting cell proliferation and inhibiting cell apoptosis^41^. The immune-related genes in this prognostic gene model are closely related to the occurrence and development of NSCLC. Related immune genes can be used as specific molecular markers for early diagnosis of NSCLC, and they can also be used as indicators for prognostic evaluation.

This study suggests that the degree of neutrophil infiltration had a certain correlation with the risk score, but there was no statistically significant difference. There was no correlation with B cells, CD4 + T cells, CD8 + T cells, dendritic cells, and macrophages. The cytoplasm of neutrophils contains a large number of neutral fine particles that are neither basophilic nor acidophilic. Most of these particles are lysosomes, containing peroxidase, lysozyme, alkaline phosphatase and acid hydrolase, etc. which are related to the phagocytic and digestive functions of cells. Neutrophil count is a representative indicator of systemic inflammation, and its increase is associated with the poor prognosis of many cancer^42^. In the tumor microenvironment, neutrophils can be manipulated, including in the early stages of the differentiation process, to develop different phenotypes and functional polarization states, thereby inducing anti-tumor or pro-tumor effects^43^. In the pro-inflammatory state, it will rapidly increase the production of neutrophils and release immature or poorly differentiated neutrophils. The recruitment of these immature neutrophils into the tumor matrix can inhibit cell apoptosis, promote metastasis and angiogenesis leading to tumorigenicity^44^. Fred Hutchinson's researchers found that neutrophils in tumors can continuously produce substances that inhibit the activity of T cells, which affects the efficacy of immune checkpoint inhibitors against tumors^45^. Recently, the neutrophil to lymphocyte ratio (NLR) has been the most extensively studied in solid tumors. Koung Jin et al. retrospectively analyzed the relevant data of 54 patients with non-small cell lung cancer treated with PD-1 inhibitors. Multivariate analysis showed that higher NLR after treatment was an independent prognostic factor for shorter PFS and OS^46^. Relevant studies have shown that neutrophils can be used as a predictor of immunotherapy response and can help make clinical decisions in specific situations. This study evaluated the correlation between the risk score of the prediction model and the penetration of six types of immune cells in the tumor microenvironment, which may provide an important reference for monitoring the status of the tumor microenvironment to guide immunotherapy.

However, this study has certain limitations. First, verifying the capabilities of the predictive model still requires a large amount of evidence-based medical evidence from multiple centers. Second, the prognostic evaluation model is based on the results of RNA sequencing analysis in the TCGA database. Third, there is a lack of clinical, cellular, and animal functional tests; hence, the reliability of data analysis results needs further verification.

## Supplementary Information

Supplementary Information 1.

Supplementary Information 2.
